# Detecting *Rickettsia parkeri* Infection from Eschar Swab Specimens 

**DOI:** 10.3201/eid1905.120622

**Published:** 2013-05

**Authors:** Todd Myers, Tahaniyat Lalani, Mike Dent, Ju Jiang, Patrick L. Daly, Jason D. Maguire, Allen L. Richards

**Affiliations:** Naval Medical Research Center, Silver Spring, Maryland, USA (T. Myers, J. Jiang, A.L. Richards);; Naval Medical Center, Portsmouth, Virginia, USA (T. Lalani, P.L. Daly, J.D. Maguire);; Naval Air Station, Pensacola, Florida, USA (M. Dent)

**Keywords:** Rickettsia, Swab, Eschar, R. parkeri, diagnostic, diagnosis, bacteria, vector-borne infections

## Abstract

The typical clinical presentation of several spotted fever group *Rickettsia* infections includes eschars. Clinical diagnosis of the condition is usually made by analysis of blood samples. We describe a more sensitive, noninvasive means of obtaining a sample for diagnosis by using an eschar swab specimen from patients infected with *Rickettsia parkeri*.

Until 2004, all confirmed cases of tick-borne spotted fever in North, Central, and South America were attributed to 1 pathogen, *Rickettsia rickettsii*, the cause of Rocky Mountain spotted fever. Historically, in the Western Hemisphere, tick-borne rickettsiae other than *R. rickettsii* were often described as nonpathogens (*1*).

In 2004, an otherwise healthy US serviceman living in the Tidewater region of eastern Virginia, USA, sought treatment at an acute care clinic with fever, mild headache, malaise, diffuse myalgias and arthralgias, and multiple eschars on his lower extremities. He reported frequent tick and flea exposures but could not recall a specific arthropod bite before illness. However, *R. parkeri,* a tick-associated *Rickettsia *species, was subsequently isolated from an eschar biopsy specimen, documenting the first recognized case of *R. parkeri* rickettsiosis (*2*,*3*). In 2006, another US serviceman visited the National Naval Medical Center with similar symptoms. He had recently returned from a vacation in the Virginia Beach area; subsequently, *R. parkeri* was also isolated from this patient (*4*). To date, >25 cases of *R. parkeri* infections have been diagnosed in the United States and South America (*5*).

*R. parkeri* was first isolated from Gulf Coast ticks (*Amblyomma maculatum*) in 1937. The organism remained relatively obscure for the next several decades. *R. parkeri* is a member of the spotted fever group (SFG) of rickettsiae, which are gram-negative obligate intracellular rod-shaped bacteria transmitted by an arthropod vector. *A. maculatum *ticks, the vectors for *R. parkeri* in the United States, have a distribution that extends across all states bordering the Gulf of Mexico and includes several other southern, mid-Atlantic, and central states (*6*). *R. parkeri* has been detected in or isolated from *A. maculatum* ticks in many of these states.

Generally, the clinical signs and symptoms of SFG rickettsioses begin 6–10 days after a person has been bitten by an infected arthropod and typically include fever, headache, myalgias, a characteristic inoculation eschar at the bite site (depending on *Rickettsia* species) (Figure), a macular or maculopapular rash, and regional lymphadenopathy (*3*). SFG rickettsioses in humans range from mild to life threatening. Rocky Mountain spotted fever is considered the most severe SFG rickettsiosis; mortality rate can be as high as 50% without adequate antimicrobial drug treatment (*7*). Death from the rickettsioses can generally be prevented if diagnosis is timely and proper treatment is given.

**Figure:**
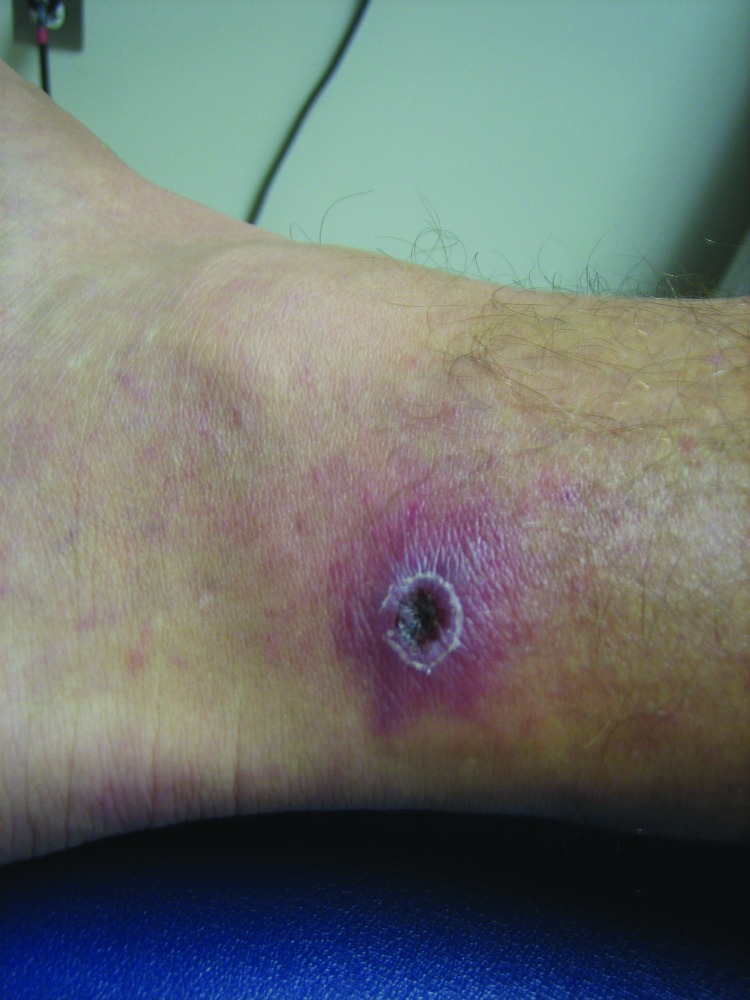
Acute eschar of patient who was subsequently diagnosed with *Rickettsia parkeri* infection in Pensacola, Florida, USA, in August 2011. This same eschar was unsheathed and swabbed after 14 days of antimicrobial drug treatment and had undergone significant healing. It still gave a positive result by real time PCR, although the convalescent-phase blood specimen showed a negative result.

## The Study 

In an ongoing, prospective study of clinical rickettsial disease in the Tidewater region of Virginia and Jacksonville, Florida, one of the primary objectives is to identify the prevalence of *R. parkeri* infection among persons seeking care with tick bite eschars or who have received a clinical diagnosis of rickettsial illness. One patient, a 43-year-old man, visited his primary care clinic in Virginia in early June 2011 after an eschar developed on his left knee, where he had removed an embedded tick 8 days previously. Topical mupirocin was prescribed for his condition. However, later that day, he experienced a fever of 104°F (40°C), accompanied by chills, night sweats, a diffuse maculopapular rash, headache, mylagias, neck stiffness, arthralgias, and malaise. He returned to the primary care clinic 3 days after onset of the fever and was prescribed a 2-week course of doxycycline. He fully recovered after the course of doxycycline.

Whole blood specimens and a swab specimen of the unroofed eschar were collected at the time he sought treatment with the acute febrile illness before doxycycline administration. Blood was collected again 25 days later during the convalescent phase. The plasma and the buffy coat were separated from the erythrocytes after centrifugation. The eschar swab was rinsed in 300 μL of phosphate-buffered saline. DNA was extracted from the buffy coat and the swab sample rinse by using QIAamp blood mini kit (QIAGEN, Germantown, MD, USA). Three microliters of the DNA preparations were applied to the *Rickettsia* genus–specific quantitative real-time PCRs (qPCRs) targeting the 17-kDa antigen gene (Rick17b) (*8*) and *gltA* (*9*), as well as the *R. parkeri* species–specific qPCR (Rpark), as described (*8*). Positive reactions were obtained from all 3 qPCRs for the swab sample and the Rick17b assay for the buffy coat. Thus, the diagnosis of *R. parkeri* infection in this patient was confirmed by molecular assays. The *R. rickettsia–*specific qPCR (Rrick) (*10*) yielded negative results.

Acute-phase and convalescent-phase plasma samples were tested for SFG-specific IgG and IgM by ELISA using *Rickettsia conorii* whole cell antigen preparation (*11*). The ELISA was performed side by side in 4-fold dilutions from 1:100 to 1:6,400. The SFG group–specific IgM was not detected in both samples, and the SFG group–specific IgG was also not detected in the acute sample but was detected in the convalescent-phase sample at a titer of 400. The serologic results confirmed the patient’s infection with a SFG rickettsial agent.

A second patient, a 36-year-old man, sought treatment at a primary care clinic in Pensacola, Florida, in late August 2011, 10 days after he was bitten by a tick. He was otherwise healthy, but a painless eschar had developed on his left ankle ≈4 days after exposure to the tick. Over the next several days, a generalized vesicular rash developed on his torso and upper and lower extremities, along with fevers >101°F, chills, night sweats, a mild headache, and generalized lymphadenopathy. The patient was prescribed a 14-day course of doxycycline at his initial visit. His symptoms improved within 24 hours. Serum samples were collected at his initial clinic visit, before initiation of antimicrobial drug therapy.

He returned to the clinic and was found to be asymptomatic after 14 days of treatment. Whole blood and a swab of his healing eschar—the crust was unroofed on sampling—were taken at his return visit. DNA was extracted from acute-phase serum and the eschar swab by using the same procedure as had been used for the first patient. Positive reactions were obtained from the swab sample by Rick17b and Rpark qPCRs, and negative by Rrick assay. The acute-phase serum sample was negative by all qPCRs mentioned above. The acute-phase serum and the convalescent-phase plasma samples were tested for SFG rickettsiae–specific IgM and IgG as was performed for the first patient. The titer of IgM for both samples was 400, whereas the IgG for the acute-phase serum and the convalescent-phase plasma samples were 1,600 and 6,400, respectively. The 4-fold raise in IgG titer between acute- and convalescent-phase samples provided the evidence for a current infection.

## Conclusions

Although *R. rickettsii* is reportedly the predominant pathogen causing human SFG disease in the United States, the actual prevalence of *R. parkeri* is unknown because most commercial serologic assays do not distinguish between SFG species. Seroprevalence studies have also demonstrated higher than expected *R. rickettsii* seropositivity among the general population without history of Rocky Mountain spotted fever, which suggests that they may have been infected by less pathogenic SFG rickettsiae such as *R. parkeri*. Recently, qPCRs have demonstrated to be effective at identifying rickettsiae specific to the species level (*12*). A report by Bechah et al. (*12*) has shown that rickettsial infection could be diagnosed from noninvasively collected cutaneous lesion swab specimens from skin eschars from guinea pigs.

Using this model and our qPCR Rick17b (*8*), we were able to obtain positive results not only from the buffy coat sample from the Tidewater patient but also a positive reaction from a swab specimen from the eschar of the same patient. In the second patient, we were able to obtain a positive reaction from the swab specimen from the healing eschar 14 days after antimicrobial drug treatment. This finding indicates that positive reaction results may be obtained from a healing eschar 14 days after antimicrobial drug treatment and is consistent with the recent report of diagnosis of SFG from swab specimens from patients in Algeria (*13*). In addition, evidence is mounting that eschars can be tested for rickettsial DNA from the point of signs and symptoms all the through the convalescent phase.
